# Cardiac Dimensions and Function are Not Altered among Females with the Myalgic Encephalomyelitis/Chronic Fatigue Syndrome

**DOI:** 10.3390/healthcare8040406

**Published:** 2020-10-16

**Authors:** Per Ole Iversen, Thomas Gero von Lueder, Kristin Reimers Kardel, Katarina Lien

**Affiliations:** 1 Department of Nutrition, Institute of Basic Medical Sciences, University of Oslo, 0317 Oslo, Norway; k.r.kardel@medisin.uio.no (K.R.K.); Katarina.Lien@follolms.no (K.L.); 2 Department of Hematology, Oslo University Hospital, 0424 Oslo, Norway; 3 Department of Cardiology, Oslo University Hospital, 0424 Oslo, Norway; tomvonoslo@yahoo.com; 4 CFS/ME Centre, Division of Medicine, Oslo University Hospital, 0424 Oslo, Norway

**Keywords:** cardiac function, echocardiography, myalgic encephalomyelitis/chronic fatigue syndrome

## Abstract

*Background:* Myalgic encephalomyelitis/chronic fatigue syndrome (ME/CFS) is a debilitating condition associated with several negative health outcomes. A hallmark of ME/CFS is decreased exercise capacity and often profound exercise intolerance. The causes of ME/CSF and its related symptoms are unknown, but there are indications of a dysregulated metabolism with impaired glycolytic vs oxidative energy balance. In line with this, we recently demonstrated abnormal lactate accumulation among ME/CFS patients compared with healthy controls after exercise testing. Here we examined if cardiac dimensions and function were altered in ME/CFS, as this could lead to increased lactate production. *Methods:* We studied 16 female ME/CFS patients and 10 healthy controls with supine transthoracic echocardiography, and we assessed cardiac dimensions and function by conventional echocardiographic and Doppler analysis as well as novel tissue Doppler and strain variables. *Results:* A detailed analyses of key variables of cardiac dimensions and cardiac function revealed no significant differences between the two study groups. *Conclusion:* In this cohort of well-described ME/CFS patients, we found no significant differences in echocardiographic variables characterizing cardiac dimensions and function compared with healthy controls.

## 1. Introduction

Myalgic encephalomyelitis/chronic fatigue syndrome (ME/CFS) is characterized by fatigue, post-exertional malaise, pain, sleeping disturbances and exercise intolerance. Although its cause remains unknown, recent data point to dysregulated energy metabolism as a possible contributing factor [1,2]. In line with this, we recently demonstrated abnormal lactate accumulation and early gas exchange threshold among ME/CFS patients compared with healthy subjects during and after exercise testing [3]. Notably, the differences in lactate accumulation and gas exchange threshold between the patients and controls increased when they were re-tested after 24 h. Neither resting nor maximum heart rate at peak exercise differed significantly between the groups [3]. Such alterations in the oxidative vs. glycolytic energy balance might be due to intrinsic abnormalities in various energy-yielding metabolic pathways and/or reduced tissue oxygen supply [1,4].

Studies with magnetic resonance imaging (MRI) of the heart have suggested lower left ventricular dimensions and provided a plausible basis for reduced left ventricular function and exercise intolerance in ME/CSF [5]. In support of this, Miwa et al. have shown that ME patients often have small hearts as measured using chest roentgenograms and cardiac dysfunction evaluated with echocardiography [6]. Corroborating this notion of impaired cardiovascular capacity in ME/CFS are data from a systematic meta-analysis showing differences in various heart-rate indices in ME/CFS compared to healthy controls [4]. Notwithstanding these findings, detailed studies of cardiac function in rigorously defined ME/CFS cohorts are scarce. We therefore assessed conventional and advanced echocardiographic variables in a well-defined cohort of ME/CFS patients and in healthy controls to examine potential differences in cardiac dimensions and function.

## 2. Materials and Methods

### 2.1. Study Approvals and Participants

Approval was obtained from the Regional Committee for Medical and Health Research Ethics in Norway (no. 2012/571-1), and the original study [3] is registered with ClinicalTrials.org (ID NCT02970240). The original cohort consisted of 18 female, normotensive ME/CFS patients of moderate severity and 15 healthy, normotensive female controls. The patients did not use any drugs regularly, and they were diagnosed with ME/CFS > 2 years prior to the study. We limited the study population to females because (i) CFS/ME is more prominent among women compared with men and (ii) we could more consistently match patients and controls. Among these, two patients and three controls did not volunteer for the heart examination, and two other controls did not attend due to logistical reasons. Hence, 16 patients and 10 controls were available for the current study. The patients fulfilled The Canadian Consensus Criteria for ME/CFS [7]. Pregnant women, those who were completely bedridden or had comorbidities and those who used heart/lung medication were excluded. The enrolment procedure and characteristics of the two study groups have been reported [3].

### 2.2. Echocardiographic Measurements

We performed supine transthoracic echocardiography (GE Vingmed E9 scanner, Horten, Norway) after an overnight fast. Conventional echocardiographic, Doppler data and strain variables were analyzed offline by a trained specialist (TGvL) unaware of group affiliation, in accordance with established guidelines [8] and using commercially available software (Echopac vers. 201, GE Healthcare). The two study groups were matched for age and body mass index (BMI).

### 2.3. Statistical Analyses

All datasets showed a normal distribution as evidenced by Q–Q plots and Shapiro–Wilk’s test. Thus, we used Student’s t test to examine differences between the two study groups. Due to multiple comparisons between the two study groups, there was a possibility of familywise error rate (and thus Type I errors). We therefore adjusted the *p*-values using the Holm–Bonferroni sequential correction method [9]. This is a less strict method than the conventional Bonferroni correction, so the chances of Type II errors are reduced. Statistical significance was set at *p* < 0.05.

## 3. Results

### 3.1. Characteristics of the Study Participants

The mean (range) ages of the ME/CFS patients and the healthy controls were 40.1 (23.9–52.0) and 35.5 (25.0–44.3) years (*p* = 0.13), respectively, and the corresponding BMIs were 24.9 (18.6–31.3) and 23.7 (18.8–35.6) kg/m^2^, respectively (*p* = 0.54). On the day of the examination, cardiorespiratory symptoms were not reported by any of the participants in either of the two study groups.

### 3.2. Echocardiographic Findings

Cardiac dimensions and function determined by echocardiography are summarized in Table 1. Here, no significant differences in systolic or diastolic left ventricular diameters were found between the ME/CFS patients and the healthy controls. Normalization of left ventricular dimensions to body surface area was also not significantly different between study groups. Moreover, the left atrial diameter was not different (*p* > 0.05) either. In line with these findings, no significant differences in systolic or diastolic blood pressure or in estimated systolic pulmonary artery pressure were found (data not shown). We next performed a wide range of assessments characterizing cardiac function in detail, both in diastole and systole. In addition to standard analysis of biplane left ventricular volumes and ejection fraction (ad modum Simpson), novel speckle-tracking echocardiography was performed. Global longitudinal left ventricular strain revealed similar results (*p* > 0.05) between the two study groups. Right ventricular function evidenced by tricuspid annulus plane systolic excursion (TAPSE) and peak systolic tissue Doppler velocity (not shown) was also comparable.

Impaired diastolic function is an established contributor to impaired exertional capacity. Here, early and late diastolic filling capacities were similar, as well as was early relaxation by tissue Doppler imaging and computed left ventricular filling pressures (i.e., E/é) (*p* > 0.05). In summary, we could not find any significant differences in any parameters of cardiac function between the ME/CFS patients and the healthy controls.

## 4. Discussion

In this study, we were not able to detect any significant differences between ME/CFS patients and healthy controls in a wide range of variables characterizing cardiac dimensions and function with the use of conventional and advanced echocardiography. These findings, therefore, do not support the hypothesis that reduced cardiac dimensions or function may contribute to early exertional lactate accumulation, early gas exchange threshold or the low exercise capacity in ME/CFS that we recently reported [3]. Our findings are similar to those reported by Montague et al. [10], but at variance with previous studies reporting smaller hearts and dysregulated autonomic regulation of cardiac function in ME/CFS [4,5,6]. Possible explanations for these discrepancies include differences in age, gender, diagnostic criteria for ME/CFS and methods for assessments of cardiac dimensions and function. For example, Miwa et al. applied the Fukuda criteria [11], whereas we used the Canadian Consensus Criteria for ME/CFS [7]. Importantly, the occurrence of post-exertional malaise is only mandatory in the latter criteria. Notably, whereas these previous reports did not correct for multiple statistical testing among MRI- and echocardiographic-derived variables, we included robust adjustments. Notwithstanding these contrasting results, those reported between ME/CFS and healthy controls in previous studies might be too small to fully explain exercise intolerance and the various metabolic abnormalities associated with ME/CSF [1,2,3].

Evidence for a reduced cardiac capacity in ME/CFS may not be evident in the resting state due to compensatory mechanisms, and absence of statistical differences in key echocardiographic parameters at rest do not necessarily mean that cardiac exertional capacities are similar. Moreover, in our previous study, we found similar blood concentrations of lactate among ME/CFS patients and controls at rest before the first exercise test, but during exercise the lactate concentration accumulated faster among the ME/CFS patients [3]. It would therefore be of interest to analyze cardiac function during exercise testing and examine whether cardiac variables are associated with markers of metabolic pathways such as enzymes and/or substrates for oxidative phosphorylation to generate ATP. For example, abnormal regulation of pyruvate dehydrogenase kinase has been associated with ME/CFS, and this could lead to an increased lactate accumulation during exercise [1].

Limitations of our study were the small sample size and that we only included female ME/CFS patients with moderate disease severity. Strengths include well-characterized participants, including a matched control group, and use of detailed, state-of-the-art echocardiography.

## 5. Conclusions

We found no significant differences in a wide range of conventional and novel echoardiographic variables characterizing cardiac dimensions and function when comparing ME/CFS patients with healthy controls. Further studies performed with exercising individuals, as well as directly linking cardiac function to biomarkers of metabolism, would be of interest.

## Figures and Tables

**Table 1:** Echocardiographic variables obtained in the ME/CFS patients and in the healthy controls.

Echocardiographic Variables	ME/CFS Patients (*n* = 16)	Controls (*n* = 10)	Crude-*p*	Adj.-*p*
**Cardiac dimensions**				
Left atrial area (cm^2^)	17.0 (3.4)	16.9 (3.4)	0.60	>0.90
Left ventricular end-diastolic diameter (cm)	4.78 (0.38)	4.70 (0.32)	0.95	>0.90
Septal wall thickness (cm)	0.78 (0.16)	0.70 (0.10)	0.17	>0.90
Left ventricular systolic diameter (cm)	3.0 (0.29)	2.86 (0.27)	0.67	>0.90
Left ventricular end-diastolic volume (mL)	91.3 (20.4)	95.0 (22.4)	0.72	>0.90
**Systolic and diastolic function**				
Heart rate (beats/min)	73 (10)	68 (14)	0.29	>0.90
Ejection fraction EF (%)	60.1 (4.6)	56.5 (3.6)	0.044	0.62
Fractional shortening (%)	35.4 (5.9)	39.2 (3.8)	0.10	>0.90
Global longitudinal strain (%)	19.1 (2.0)	19.9 (1.0)	0.33	>0.90
Early transmitral flow (E; m/s)	0.67 (0.14)	0.66 (0.08)	0.86	>0.90
Atrial transmitral flow (A; m/s)	0.46 (0.12)	0.41 (0.16)	0.35	>0.90
Ratio of early and atrial transmitral flow (E/A)	1.55 (0.56)	1.85 (0.62)	0.21	>0.90
Pulsed tissue Doppler (e’; cm/s)	11.6 (1.9)	13.6 (2.4)	0.046	0.62
Ratio of early transmitral flow and e’ (E/e’)	5.96 (1.14)	5.06 (1.16)	0.10	>0.90
Tricuspid annular plane systolic excursion (cm)	5.0 (8.0)	2.4 (0.4)	0.31	>0.90
Stroke volume (mL)	59.5 (9.4)	62.3 (9.2)	0.49	>0.90
Stroke volume index (mL/m^2^)	24.3 (15.1)	36.0 (4.7)	0.0094	0.14
Cardiac output (L/min)	4.3 (0.7)	4.2 (0.9)	0.74	>0.90
Cardiac index (L/min/m^2^)	1.8 (1.1)	2.4 (0.4)	0.098	>0.90

Values are mean (SD). Crude-*p*, unadjusted *p*-values; Adj.-*p*, *p*-values adjusted according to the Holm–Bonferroni sequential method.

## References

[1] Fluge O., Mella O., Bruland O., Risa K., Dyrstad S.E., Alme K., Rekeland I.G., Sapkota D., Røsland G.V., Fosså A. (2016). Metabolic profiling indicates impaired pyruvate dehydrogenase function in myalgic encephalopathy/chronic fatigue syndrome. JCI Insight.

[2] Tomas C., Brown A., Strassheim V., Elson J.L., Newton J., Manning (2017). P. Cellular bioenergetics is impaired in patients with chronic fatigue syndrome. PLoS ONE.

[3] Lien K., Johansen B., Veierod M.B., Haslestad A.S., Bøhn S.K., Melsom M.N., Kardel K.R., Iversen P.O. (2019). Abnormal blood lactate accumulation during repeated exercise testing in myalgic encephalomyelitis/chronic fatigue syndrome. Physiol. Rep..

[4] Nelson M.J., Bahl J.S., Buckley J.D., Thomson R.L., Davison K. (2019). Evidence of altered cardiac autonomic regulation in myalgic encephalomyelitis/chronic fatigue syndrome: A systematic review and meta-analysis. Medicine.

[5] Olimulder M.A., Galjee M.A., Wagenaar L.J., van Es J., van der Palen J., Visser F.C., Vermeulen R.C.W., von Birgelen C. (2016). Chronic fatigue syndrome in women assessed with combined cardiac magnetic resonance imaging. Neth. Heart J..

[6] Miwa K., Fujita M. (2009). Cardiovascular dysfunction with low cardiac output due to a small heart in patients with chronic fatigue syndrome. Intern. Med..

[7] Carruthers B., Jain A.K., De Meirleir K.L., Peterson D.L., Klimas N.G., Lerner A.M., Bested A.C., Flor-Henry P., Joshi P., Powles P. (2003). Myalgic Encephalomyelitis/Chronic Fatigue Syndrome. J. Chron. Fatigue Syndr..

[8] Lang R.M., Badano L.P., Mor-Avi V., Afilalo J., Armstrong A., Ernande L., Flachskampf F.A., Foster E., Goldstein S.A., Kuznetsova T. (2015). Recommendations for cardiac chamber quantification by echocardiography in adults: An update from the American Society of Echocardiography and the European Association of Cardiovascular Imaging. J. Am. Soc. Echocardiogr..

[9] Holm S. (1979). A simple sequential rejective method procedure. Scand. J. Stat..

[10] Montague T.J., Marrie T.J., Klassen G.A., Bewick D.J., Horacek B.M. (1989). Cardiac function at rest and with exercise in the chronic fatigue syndrome. Chest.

[11] Fukuda K., Straus S.E., Hickle I., Sharpe M.C., Dobbins J.G., Komaroff A. (1994). The chronic fatigue syndrome: A comprehensive approach to its definition and study. International Chronic Fatigue Syndrome Study Group. Ann. Intern. Med..

